# Dosimetric impact of rotational errors in trigeminal neuralgia radiosurgery using CyberKnife

**DOI:** 10.1002/acm2.14238

**Published:** 2023-12-22

**Authors:** Ming Liu, Joanna E Cygler, David Tiberi, Janice Doody, Shawn Malone, Eric Vandervoort

**Affiliations:** ^1^ Department of Medical Physics The Ottawa Hospital Cancer Center Ottawa Ontario Canada; ^2^ Department of Physics Carleton University Ottawa Ontario Canada; ^3^ Department of Radiology University of Ottawa Ottawa Ontario Canada; ^4^ Department of Radiation Oncology The Ottawa Hospital Cancer Centre Ottawa Ontario Canada; ^5^ Department of Radiation Oncology University of Ottawa Ottawa Ontario Canada; ^6^ Radiation Medicine Program The Ottawa Hospital Cancer Centre Ottawa Ontario Canada

**Keywords:** CyberKnife, rotations, stereotactic radiosurgery, trigeminal neuralgia, uncertainty modeling

## Abstract

**Purpose:**

Trigeminal neuralgia (TN) can be treated on the CyberKnife system using two different treatment delivery paths: the general‐purpose full path corrects small rotations, while the dedicated trigeminal path improves dose fall‐off but does not allow rotational corrections. The study evaluates the impact of uncorrected rotations on brainstem dose and the length of CN5 (denoted as L_eff_) covered by the prescription dose.

**Methods and materials:**

A proposed model estimates the delivered dose considering translational and rotational delivery errors for TN treatments on the CyberKnife system. The model is validated using radiochromic film measurements with and without rotational setup error for both paths. L_eff_ and the brainstem dose is retrospectively assessed for 24 cases planned using the trigeminal path. For 15 cases, plans generated using both paths are compared for the target coverage and toxicity to the brainstem.

**Results:**

In experimental validations, measured and estimated doses agree at 1%/1 mm level. For 24 cases, the treated L_eff_ is 5.3 ± 1.7 mm, reduced from 5.9 ± 1.8 mm in the planned dose. Constraints for the brainstem are met in 23 cases for the treated dose but require frequent treatment interruption to maintain rotational corrections <0.5° using the trigeminal path. The treated length of CN5, and plan quality metrics are similar for the two paths, favoring the full path where rotations are corrected.

**Conclusions:**

We validated an analytical model that can provide patient‐specific tolerances on rotations to meet plan objectives. Treatment using the full path can reduce treatment time and allow for rotational corrections.

## 1 INTRODUCTION

Stereotactic radiosurgery (SRS) can be used to treat trigeminal neuralgia (TN), a rare nerve disorder that causes severe, debilitating facial pain.^1^ During SRS treatment for TN, radiation beams are precisely directed at a segment of cranial nerve 5 (CN5), which is also referred to as the trigeminal nerve. These treatments require a high degree of spatial accuracy to treat the very small target, typically a 3 mm wide, 4 to 10 mm length of CN5.^2^ The CyberKnife G4 and VSI model robotic radiosurgery system by Accuray Inc., Sunnyvale, CA, make use of a specialized TN treatment mode. Although TN treatment mode has been discontinued in M6 or higher models, there are still at least 116 older units in clinical use around the world (The IAEA Directory of Radiotherapy Centres (DIRAC), https:/dirac.iaea.org/ (accessed on May 24, 2023). In TN mode, the linac is brought closer to the patient than other cranial sites to more efficiently deliver the large single dose of radiation required. During this time, the target position (translation and rotation) is tracked by acquiring planar orthogonal X‐ray images of the patient's skull every 20−30 s.^3^ For the TN treatment mode, the beam source positions and the path that the robot follows between beams are referred to as the trigeminal path. The beam directions are optimized to spare nearby radiosensitive structures such as the brainstem and temporal lobe. However, rotational corrections *cannot* be applied in TN mode without the risk of collisions. As a result, uncorrected rotations could reduce the treatment efficacy and increase the toxicity to organs at risk. Alternatively, for other cranial treatments, a different set of beams and path (referred to as the full path) is used and the CyberKnife robot arm can adjust the direction of the beams to account for small rotations. Specifically, at our institution, for the VSI version of the CyberKnife system is used, with the standard treatment couch. For cranial treatment using the full path, this version of the CyberKnife system can correct target rotations within 1°, 1°, and 3° in roll, pitch, and yaw, respectively.

Several studies have investigated technical or anatomical factors which could impact complications and treatment outcomes for TN.^2^, ^4^, ^5^, ^6^ Du et al. found that the brainstem dose was affected by the spatial orientation of CN5 relative to the brainstem, the distance between the isocentre and brainstem, and radiation field size for 40 patients treated on a conventional linac.^2^ Flickinger et al. found that increasing the length of the nerve included in the treatment volume did not significantly improve pain relief for 87 patients treated on the Gamma Knife (Elekta AB, Stockholm, Sweden) system.^4^

Conti et al. found that multiple sclerosis, integral dose, target volume and mean dose were factors associated with pain relief durability and sensory disturbance following treatment for 296 cases treated on CyberKnife.^6^ Villavicencio et al. reported on a multi‐center study with 95 patients treated with a median minimum dose of 60 Gy (median maximum: 75 Gy) using CyberKnife.^7^ In their study, 67% experienced initial pain relief, and 50% continued pain relief in a two‐year follow‐up. Romanelli et al. have reported on relatively large cohorts of patients who received TN treatment on CyberKnife using a consistent protocol at a single center.^8^, ^9^ In Romanelli et al., 2018, the actuarial pain control rates at 6, 12, 24, and 36 months were reported as 94%, 86%, 80%, and 76%, respectively, for 138 patients with a median dose of 60 Gy to 80% isodose line. Less than 0.03 cc of the brainstem was planned to receive 15 Gy (denoted as V15Gy ≤ 0.03 cc), with an acceptable risk of hypoesthesia following retreatment. In Romanelli et al., 2019, they reported on 560 treatments for 527 patients with more than three years of follow‐up for 387 patients. A six‐millimeter segment of the nerve was treated to 60−65 Gy initially (prescribed to 80−90% isodose line) and received a further 40−45 Gy if retreatment was required. However, 49% of patients had only 4–5 mm of nerve covered due to a shorter cisternal segment of the trigeminal nerve. As mentioned previously, for their plans they used the full path, where the CyberKnife robot could correct for rotations. It was stated that the average treatment duration was 50 min. They reported that 12.8% of cases required retreatment with a pain relief rate of 76% for 343 patients within the follow‐up period. In their patient cohort, 6% of patients developed facial hypoesthesia three years post‐treatment, and most of them had this side effect after their retreatment.

At our clinic, the trigeminal path is used with rotational corrections kept to less than 0.5° based on geometric considerations. This results in longer treatment times, up to two hours long, due to more frequent adjustments of the patient's position to minimize the impact of uncorrected rotations. We developed an analytical model to estimate the dose delivered which accounts for uncorrected target rotations to answer the following questions. What is the length and volume of CN5 receiving the prescription dose if uncorrected rotations are taken into account? What differences are observed in the delivered dose to the target and brainstem for plans made using the full and trigeminal paths? In this study, we validate the dose estimated by the model using radiochromic films, evaluate the dosimetric impacts of delivery errors on the target and brainstem, and conduct a planning study to compare the plans created using two path sets.

## 2 METHODS

### 2.1 Treatment data

The contours, planned dose, and treatment delivery log files were extracted for 24 plans (CyberKnife delivery system versions 9.0‐10.5; planning system versions 3.5‐5.2). The entire length of CN5 from the sensory root entering the brainstem to the gasserian ganglion was contoured for these treatments. All data were collected as a part of the Research Ethics Board‐approved quality assurance and improvement study. The contours were created using MR images or a CT cisternogram if required.^2^, ^10^, ^11^

The typical prescription (denoted as Rx) is 60 Gy in a single fraction prescribed to the 75−80% isodose line. The target is an approximately 6 mm length of CN5 near the sensory root excluding parts of the nerve less than 2 or 3 mm from the brainstem. For three patients with recurrent pain or poor response, a retreatment dose of 50 Gy was prescribed. At our institution, the planning goals for the brainstem are V15Gy ≤ 0.03 cc, V12Gy ≤ 0.3 cc and V10Gy ≤ 1.00 cc. The 15 Gy constraint,^12^, ^13^ is stricter near maximum dose than those in other studies.^9^, ^14^, ^15^

**FIGURE 1:**
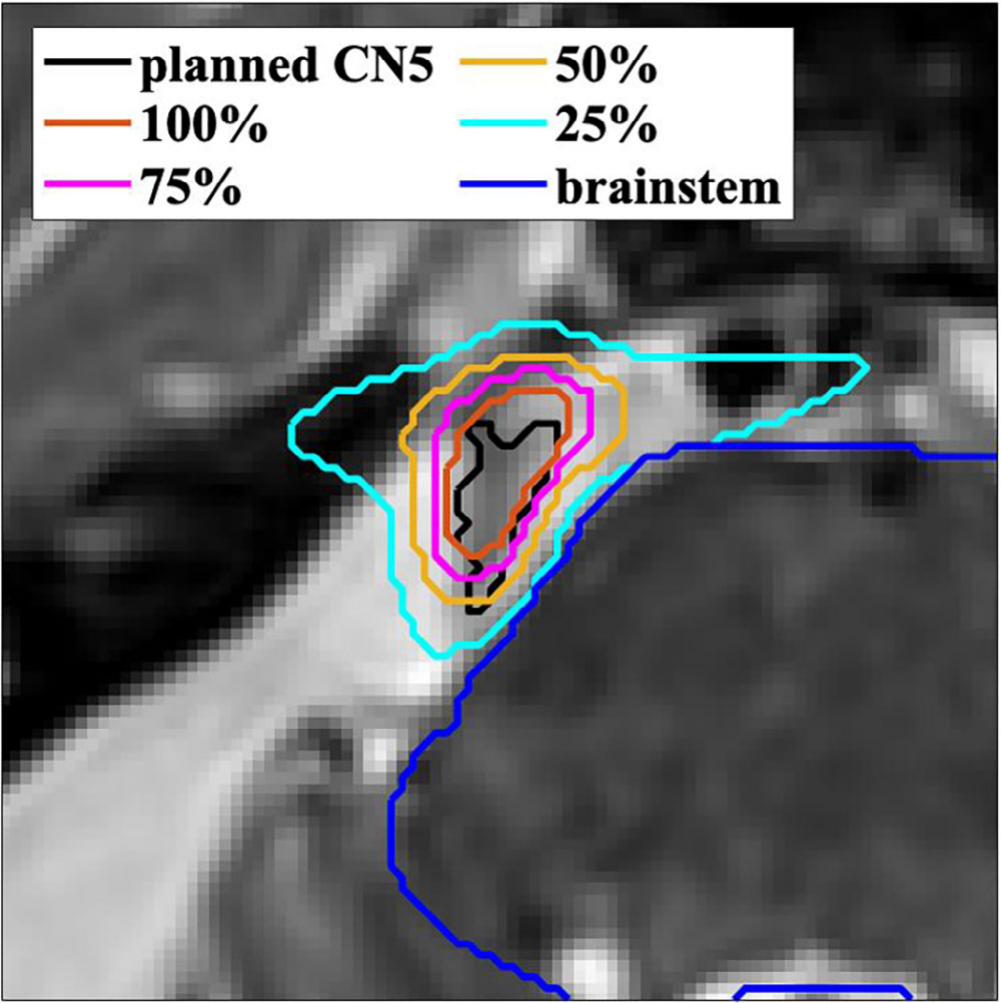
The isodose lines and contours of the trigeminal nerve (CN5) and the brainstem are shown on a SPACE T2‐weighted MR image (transverse plane). The pixel size is 0.5 mm × 0.5 mm, and the dose falls from 100% to 25% of the prescription dose within a few millimeters.

Based on geometrical considerations, to reduce the impact of uncorrected rotational errors, at our center we maintain rotations about any axis to be ≤x0.5° for treatment using the trigeminal path. This results in significantly longer treatment times (96 ± 36 min) compared to the estimated times (54 ± 7 min) provided by the planning system for our patient cohort. In Figure 1, the isodose lines and contours of CN5 and the brainstem are shown on a SPACE T2‐weighted MR image. It illustrates a steep dose gradient which falls from 100% to 25% Rx (typically 15 Gy) over just a few millimeters.

### 2.2 Dose perturbation model

**FIGURE 2:**
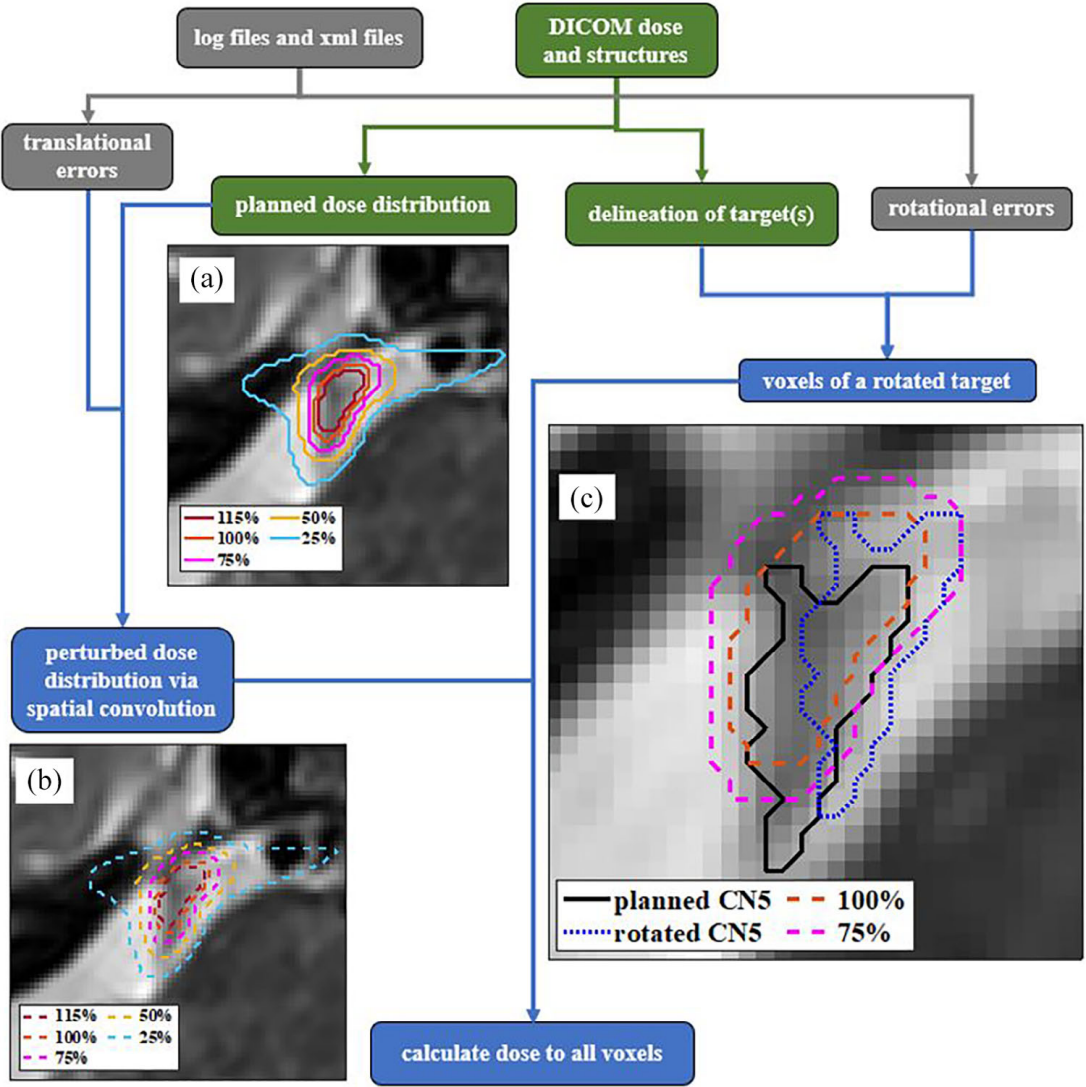
The workflow of the dose estimation model for trigeminal neuralgia treated using robotic radiosurgery. The isodose lines of the planned dose (a) and of the perturbed dose (b) are shown on a transverse T2‐weighted SPACE MR image where CN5 appears dark and the nerve passes through the hyperintense CSF. The brainstem is adjacent in the lower right. The perturbed dose considers small translational errors in beam delivery. In (c) the contours of the planned trigeminal nerve (CN5) and the rotated CN5, and the isodose lines are shown. The rotated CN5 contour shown is obtained by applying a hypothetical 0.5° rotation about the transverse plane.

In Figure 2, the workflow for the dose perturbation model is illustrated. Translational and rotational tracking errors were extracted from treatment delivery log files. The translational errors have two components. One is calculated as the difference between the translational corrections from one image to the next and is mostly <1 mm. These data approximate the distance a patient moves within their immobilization in the time between two X‐ray acquisitions. The other is a beam positioning error based on end‐to‐end quality assurance measurements for the trigeminal path.^16^ In our dose perturbation model, treatment translational uncertainties are approximated as a convolution of the planned dose with the probability density functions of these discretized translational errors. To model target rotation, a rotation matrix is applied to each target voxel position and then discretized using the nearest neighbor approximation. The perturbed dose at the rotated position is sampled from the dose matrix convolved using the translational errors. The accumulated dose in each voxel is equally weighted for every perturbation. For the trigeminal path, rotational errors are recorded per X‐ray image acquisition and are not corrected, and they are incorporated in the rotation matrix to estimate the dosimetric impact of the target rotation. In‐house MATLAB (2018b, MathWorks Inc., Natick, MA) scripts were implemented for this model.

### 2.3 Experimental validation

We validate the model by comparing the perturbed dose with film measurements in a bullet‐shaped phantom made primarily of solid water. Six fiducials are embedded to enable accurate rotational corrections.^17^, ^18^ Four Gafchromic™ EBT3 (Ashland Inc., Covington, KY) films (10.2 × 12.7 cm^2^) are used in each experiment to reduce the relative dosimetric uncertainty of measurements to within 1%.^19^, ^20^ For each experimental film, two additional reference films are cut from the same sheet. One film is irradiated with the reference field (60 mm diameter at 15 mm depth in solid water) the other is unexposed film but kept with the exposed films. Film intensities are rescaled to give correct nominal dose for the reference field and zero dose reading following Lewis et al.^21^ For film processing, we use an Epson (Epson, Nagano, Japan) 10000XL scanner (48‐bit color, 150 DPI) in transmission mode, and process data using the green colour channel. Three holes are punched in each film which fit into pins in a scanner template to place the film in the center of the scanner and only fit in one orientation. The scanner template lifts the film off scanner glass, fixed from below by the 3 pins, and by a plastic plate above which holds films flat and parallel to the scanner glass. Each film is scanned three times and averaged. Each scan includes the experimental film and the two reference films from the same sheet. We follow a slightly modified version of Devic et al.’s approach to linearize the dose‐response curve,^22^ with the exponent in the numerator adjusted from 2/3 to 4/5 which maximizes the R^2^ of the fit function for the film batch used.

The spatial registration of the films to the planned dose is based on the three holes punched in the films which are indexed in the phantom using high density pins which can be automatically identified in the films scans and CT images of the phantom, with estimated total spatial uncertainty ≤0.5 mm. They are positioned on the coronal plane of the phantom, and the measured dose is averaged over all films. Two treatment plans are generated for one of the patients in the study, one using the trigeminal path and the other using the full path. The plans are delivered to the phantom in quality assurance mode with rotational offsets of −0.6°, 0.6°, and −2.5° in roll, pitch, and yaw, respectively. The selection of a larger rotational offset, closer to the correction limits of the full path was intended to validate the model's performance when attenuation changes in tissue density and physical path length were greater when large uncorrected rotations are present. For both plans, beam weights are adjusted to give a maximum film dose of 10 Gy. The agreement between the planned (or perturbed) and measured doses is assessed using the global gamma index with a 1%/1 mm criterion. This strict gamma criterion is to emphasize the difference in dose distribution between scenarios with and without rotational corrections.^23^ The gamma pass rates were calculated for all voxels of the averaged film plane using an in‐house written MATLAB (2018b, MathWorks Inc., Natick, MA) script based on the fast 2D calculation of Wendling et al.^24^ In one of our previous studies, the model underwent a comprehensive evaluation, where ten radiochromic film measurements were conducted under nine experimental conditions.^25^

### 2.4 Quantitative metrics

We evaluate the volume and length (denoted as L_eff_) of CN5 covered by Rx isodose and dose‐volume metrics relevant to the brainstem (V10Gy, V12Gy, V15Gy) for every plan. This was done by performing principal component analysis on the coordinates of voxels within CN5. The length of the nerve is calculated as the full width at the tenth maximum of the distribution of the first principal component (denoted as PC1). The effective length (denoted as L_eff_) of CN5 receiving the Rx is calculated by counting the number of nerve cross‐sections (in PC2 and PC3) where more than half the voxels in a cross section receive equal to or greater than Rx.

### 2.5 Planning study

We replanned fifteen cases using the full path with the same plan optimization settings and parameters used in the original trigeminal path plans. Both the original and new planned dose distribution were rescaled to meet the following criteria: target D_min_ ≥ Rx if the Rx can be prescribed to ≥ 70% isodose line and the brainstem V15Gy ≤ 0.03 cc.

The dosimetric impact of rotational and translational delivery errors extracted from each patient's log files was assessed using the dose perturbation model described previously. The rotational errors were set to be the difference between two consecutive X‐ray images for the full‐path plans, and used in the rotation matrix of the dose perturbation model to evaluate the dosimetric impact of target rotations. We have confirmed that this approach is consistent with the transformation matrices used for rotational corrections applied by the system.

The gradient index is calculated for every plan as the ratio of volume receiving 50% Rx to that receiving 100% Rx.^26^ Paired sample *t*‐tests are calculated for statistical significance (*P* < 0.05) in the mean difference between these plans for several dosimetric quantities.

## 3 RESULTS

### 3.1 Experimental validation

**FIGURE 3:**
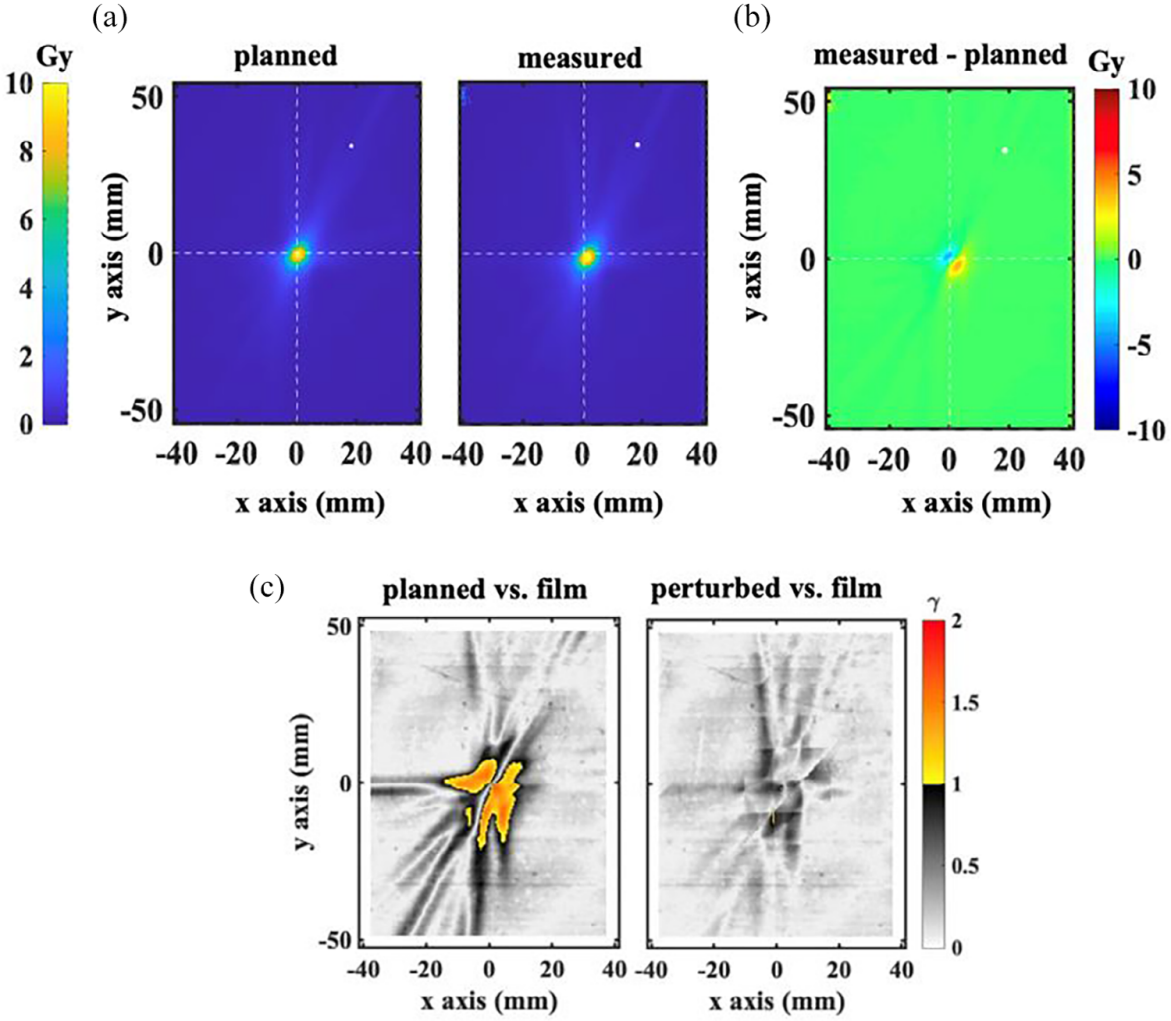
(a) The planned and measured doses. The dose was delivered using the trigeminal path, for which a rotational offset is not compensated. The films remained static during beam delivery and were positioned with a rotational offset of −0.6°, 0.6°, and −2.5° in roll, pitch, and yaw, respectively. The white dot indicates the projection of the imaging isocenter onto the film plane, about which the reported rotation is calculated. (b) The difference between the measured and planned doses. (b) Two‐dimensional gamma analyses (1%/1 mm criteria) on planned and measured dose distributions (left) and perturbed and measured dose distributions (right). The gamma pass rates for all non‐zero pixels are 96.48% and 99.98%, respectively.

Film experiment data is shown in Figure 3 for the plan using the trigeminal path. The subtracted dose image (Figure 3b) illustrates how relatively small uncorrected rotations can lead to significant underdose for small targets. Figure 3c illustrates how the perturbed dose models the impact of an uncorrectable rotation. Failures of the 1%/1 mm γ criteria (shown as the yellow to red color wash) occur in relatively large regions in and around the target. For the full path, data is not shown because the 1%/1 mm γ criteria pass rate is 100.00% for all voxels within the film plane where the planned dose is non‐zero for both the planned and perturbed dose comparison.

### 3.2 Dosimetric impacts

**FIGURE 4:**
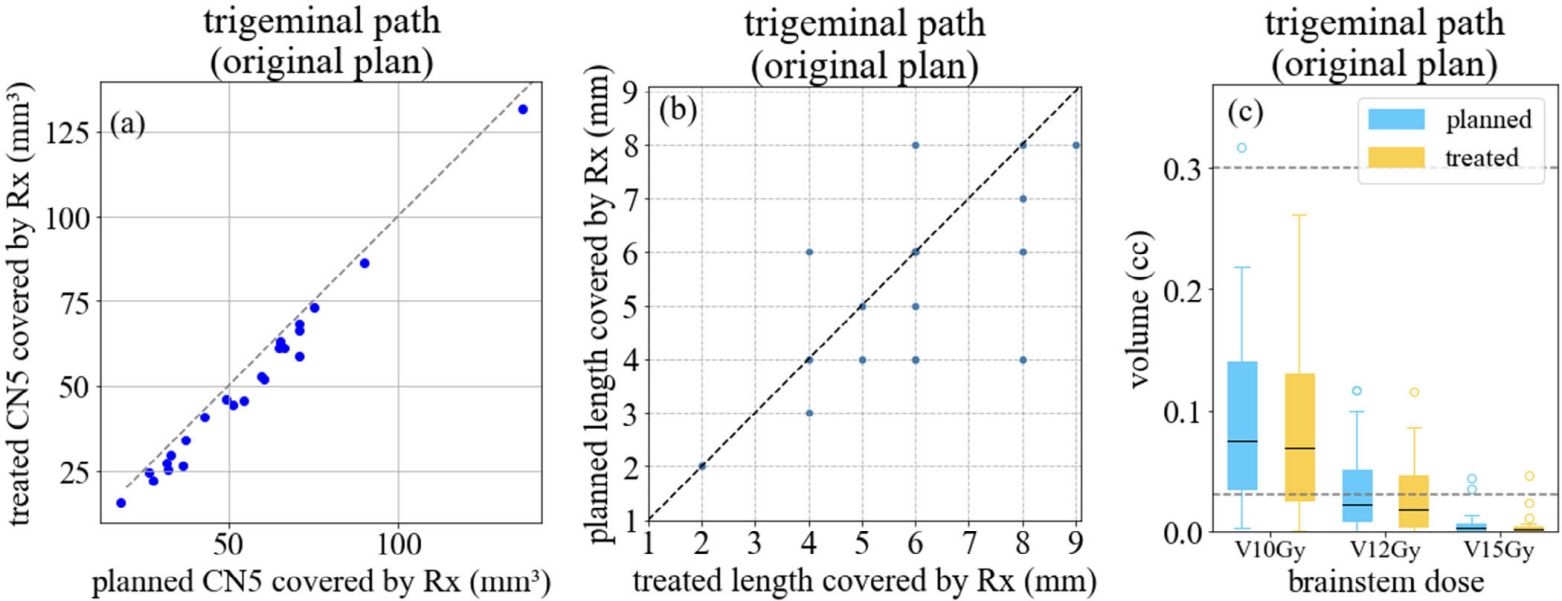
(a) The volume of the trigeminal nerve (CN5) covered by the prescription dose (Rx) for 24 cases for the original planned and the treated doses (perturbed based on delivery errors). (b) The relationship between the treated and planned nerve length covered by Rx. Any point on the reference (dashed) line indicates that the treated length is not affected by delivery errors. (c) Brainstem dose‐volume metrics. The dashed reference lines indicate the brainstem volumes of 0.03 and 0.3 cc. Our planning goals for the brainstem are V15Gy ≤ 0.03 cc, V12Gy ≤ 0.3 cc and V10Gy ≤ 1.0 cc.

Figure 4 summarizes the dosimetric impact of the observed delivery errors for 24 plans using the trigeminal path. Except for two cases treated before 2013, all patients had rotations <0.5° during treatment. Figure 4a shows the volume encompassed by Rx for the perturbed dose (denoted as “treated”) versus that (denoted as “planned”) in the original plan. This volume is reduced from 47 ± 26 mm^3^ to 43 ± 26 mm^3^ on average for the treated dose compared to the planned. Figure 4b shows the relationship between the treated and planned length of nerve covered by Rx (denoted as L_eff_). The planned L_eff_ is 5.9 ± 1.8 mm, which is reduced to 5.3 ± 1.7 mm in the treated dose. Figure 4c shows treated and planned dose‐volume metrics for the brainstem. Only one plan had V15Gy > 0.03 cc when delivery errors were considered. However, for this case, it already exceeded tolerance with brainstem V15Gy = 0.04 cc for the planned dose.

### 3.3 Planning study

**FIGURE 5:**
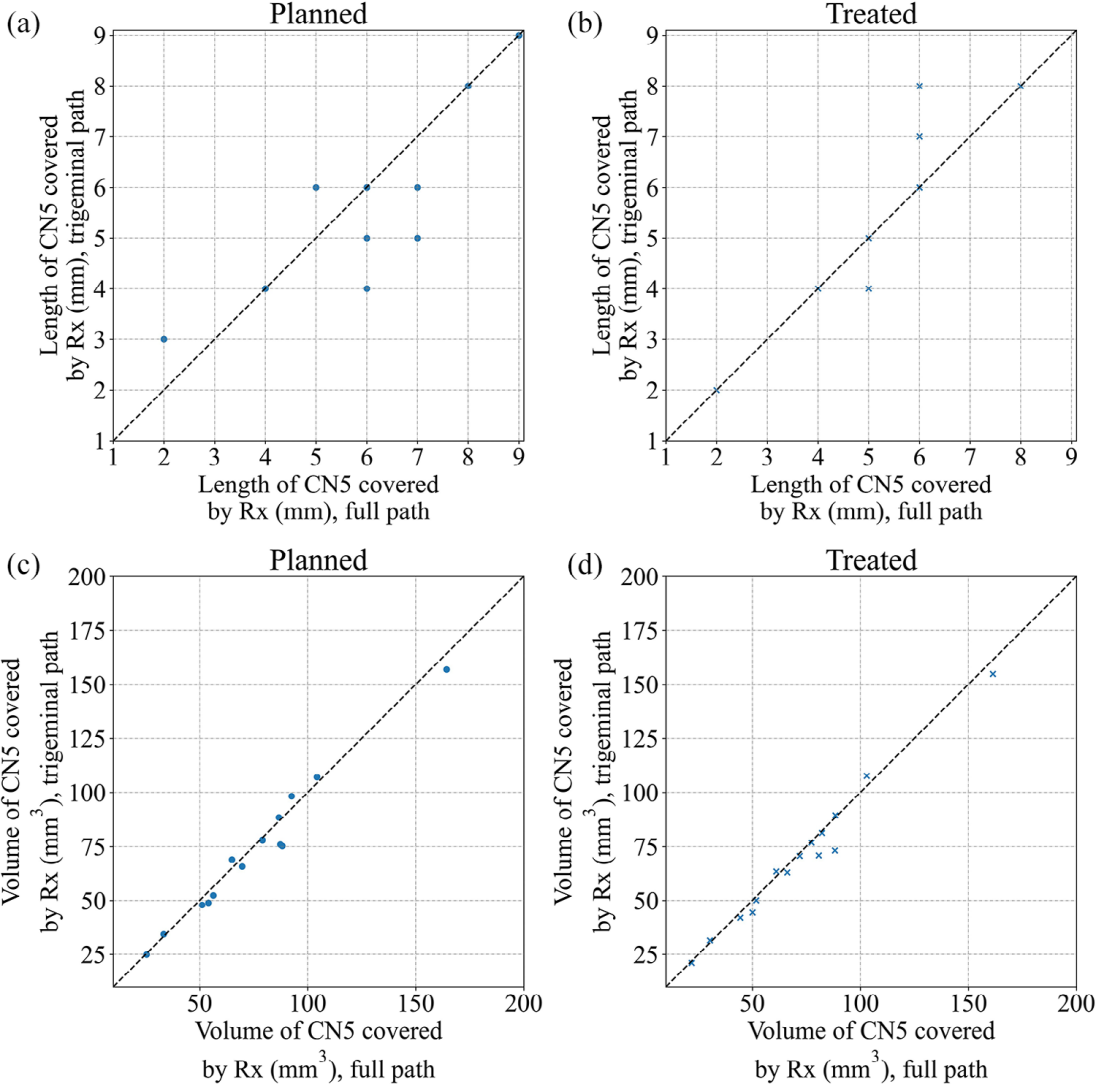
The relationship between lengths (a, b) and volumes (c‐d) of CN5 encompassed by Rx is shown at treatment planning (denoted as “planned”) and after taking into account delivery errors (denoted as “treated”) for plans created using the trigeminal path and the full path for fifteen treatments. Points on the dashed reference line indicate that the volume or length of CN5 covered by the prescription unaffected for any selected path.

For plans generated using the full path and trigeminal path, no significant difference is found in the mean difference between the Dmax/Rx, Dmin/Rx, planned length and treated length covered by Rx, and brainstem dose metrics (V10Gy, V12Gy, and V15Gy). As shown in Figure 5b, the treated L_eff_ is 5.7 ± 2.2 mm and 5.7 ± 2.1 mm for the trigeminal path plans and the full path plans, respectively. The difference in the treated and planned volume covered by Rx between the two sets of plans are shown Figure 5c‐d.

**FIGURE 6:**
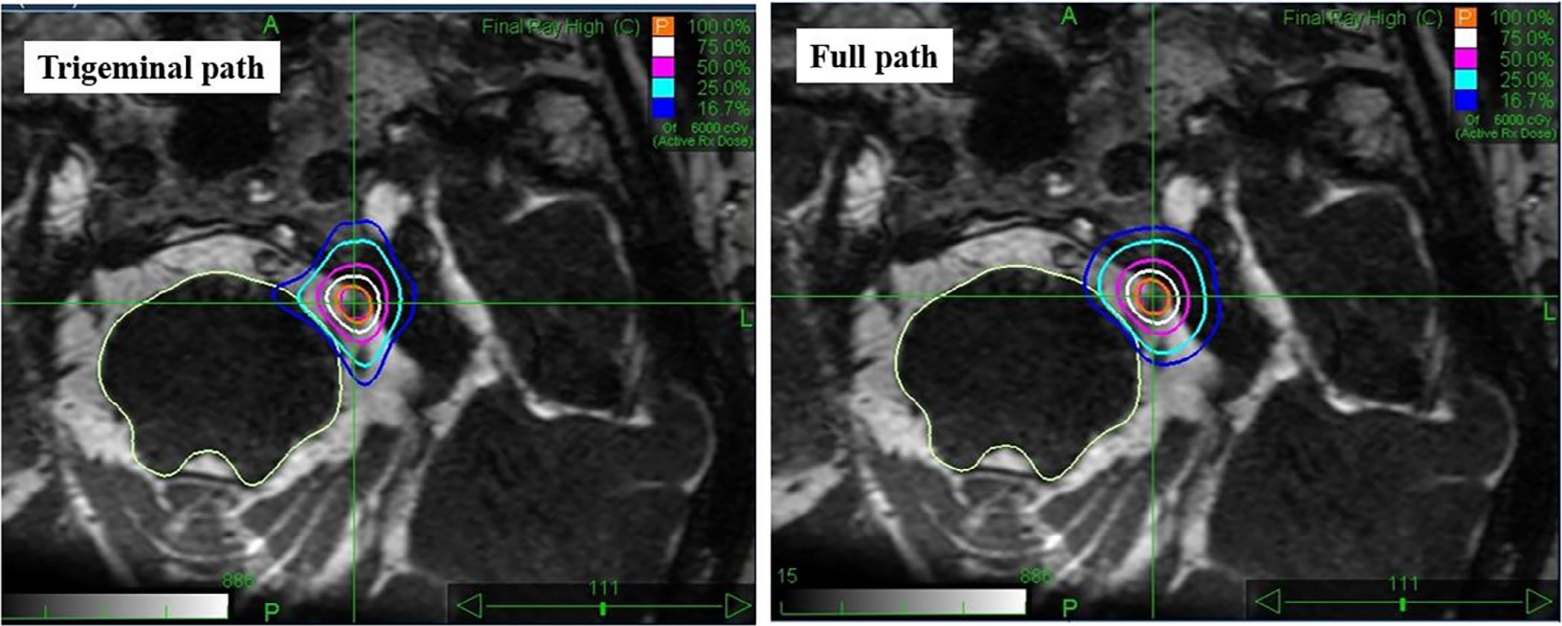
The dose distributions planned using the trigeminal path (gradient index = 5.4) and full path (gradient index = 5.9) for the same patient on a SPACE T2‐weighted MR image.

The gradient indexes are 5.4 ± 0.4 and 5.6 ± 0.5, respectively, for the trigeminal path and full path plans. The dose gradient is likely higher using the trigeminal path because the linac is brought closer to patients for dose delivery, which effectively reduces the diameter and penumbra of the beams. In Figure 6, the dose distributions created using the two path sets for the same patient are shown on a SPACE T2 weighted MR image (transverse plane). Using the trigeminal path, the dose gradient is higher, and the dose distribution is shaped around CN5 to spare the brainstem.

## 4 DISCUSSION

We validate an analytical model which estimates the perturbed dose considering uncorrected rotation and translational errors (Figure 3c). Using this model, we evaluate the volume and length of CN5 covered by Rx for both planned and treated doses. To our knowledge, this is the first study of the impact of delivery errors on radiosurgical TN treatments. Previous studies of the impact of patient motion on the target dose have primarily focused on translational uncertainties, neglecting target rotations. These studies have utilized techniques like dose‐shift via convolution,^27^, ^28^ beam‐shift via perturbing the isocenter,^28^, ^29^ and fluence convolution.^30^, ^31^ Some researchers, such as Liu et al.^32^ (one of our previous studies) and Chan et al.,^18^ have estimated dosimetric effects of translational and rotational errors for patients treated for liver lesions on CyberKnife, which employs a model for motion tracking. They assessed the dosimetric effects of rotational errors, specifically, by rotating the contours or the planning dose distribution to evaluate the target dose. However, most studies fail to account for target rotation. In contrast, our study addresses this limitation by introducing a tool that evaluates the treated length of CN5 while considering both translational and rotational motion tracking uncertainties. A similar approach could be applied to evaluate the impact of uncorrected rotation and translation errors observed for six degree of freedom surface‐guided intrafraction motion monitoring for SRS on conventional linacs. The analysis presented also expands beyond the conventional emphasis on the volume and length of CN5 covered by the prescription dose during treatment planning.^6^, ^9^ Although the sample size is small, this study demonstrates the feasibility of the approach and suggests that maintaining rotational corrections under 0.5° is safe for CyberKnife treatments using the trigeminal path. This tool provides a more realistic estimate of brainstem dose, and the volume/length of the nerve treated using this modality which may better correlate with neurotoxicity and pain relief. One limitation of this method for assessing residual error in dose delivery, is that the size of the errors assessed is comparable to the uncertainties associated with translational and rotational corrections using the skull tracking algorithm for the CyberKnife system.^3^ These uncertainties have been estimated to be on the order of ± 0.3 degrees and ± 0.5 mm, when using fiducial tracking is employed as a gold standard.^3^ The model is also limited due to the use of a nearest neighbor approximation combined with the finite dose matrix voxel size (dimensions typically between 0.5 and 1 mm).

The treatment times are on average 96 ± 36 min using the trigeminal path for our patient cohort. Romanelli et al. used the full path where the CyberKnife robot could correct for rotations.^9^ The reported treatment durations were on average 50 min (no range or standard deviation reported). They allowed a slightly higher brainstem dose and treated smaller target volumes. The brainstem V18Gy was 0.035 cm^3^ and the median target volumes were 27 ± 17 mm^3^, respectively. They reported that 49% of patients (169/343) had only 4–5 mm of nerve covered due to a shorter cisternal segment of the trigeminal nerve. In comparison, the average target volume is 48 ± 22 mm^3^ (median = 45 mm) and the planned target length is 6 ± 2 mm in our study.

The possibility of permitting larger rotations (i.e., >0.5°) to shorten the treatment time using the trigeminal path is highly patient‐dependent. Several parameters influence the reduction in volume and length of nerve treated to the prescription, including the magnitude of rotations, the isocenter to nerve distance, the dose gradient, and the target to brainstem distance. However, the dose perturbation tool could assess plan robustness to uncorrected rotations as a quality assurance test prior to each patient's treatment. We estimate that this calculation using a suitable range of permutations of roll, pitch, and yaw angles (−1.0° to 1.0° in steps of 0.5° for a total of 125 possible permutations) would require approximately 75 min (Intel Core i7‐ 8550U CPU at 1.80 GHz).

In the planning study, we compare two sets of plans using two different path sets with the same minimum dose coverage and toxicity to the brainstem and found that the treated length is very similar (Figure 5b). No statistically significant difference between the dose‐volume metrics considered is found. However, the treated volume and length of CN5 are slightly higher using the full path. Therefore, whether to use the trigeminal path for planning is a trade‐off between a few considerations: treatment time, the magnitude of rotations, the dose gradient, and the length and volume of CN5 covered by Rx. Some cases have a smaller region of cerebrospinal fluid between the brainstem and gasserian ganglion and hence a smaller length of CN5 which can be treated safely. This suggests that the full path should be considered when TN planning for small targets. For example, one case in our cohort had only 2 mm of CN5 covered by the Rx during planning. This patient was treated for over two hours with many treatment interruptions whenever the patient's rotations we beyond 0.5°. In such a case, it is preferable to correct for rotation using the full path to reduce the risk of insufficient coverage in the treated length and shorten the treatment time. Another case had 8 mm of CN5 covered by Rx during planning and was more robust to rotational error. Since the trigeminal path provides a higher dose gradient and there is sufficient length coverage, we can use the trigeminal path and allow a larger rotation (1°) to reduce the treatment time.

## 5 CONCLUSION

For small SRS targets within a high dose gradient, a relatively small uncorrected rotation can make a significant difference in the delivered dose. We validated an analytical model that can be used to evaluate the plan's robustness to delivery error prior to treatment and provide the patient‐specific tolerances on rotations that meet treatment plan objectives. We assessed the treated length and volume of the nerve and the brainstem dose considering delivery errors. A rotational correction threshold of 0.5° is a reasonable and conservative threshold for CyberKnife treatments using the trigeminal path. However, treatment using the full path should be considered to reduce treatment time and improve delivery accuracy by allowing for rotational corrections.

## AUTHOR CONTRIBUTIONS

Ming Liu: Conceived and designed the study, analyzed the data, and wrote the manuscript. Joanna Cygler: Contributed to the study design, collected and interpreted the data, and revised the manuscript. David Tiberi: Contributed to the study design and revised the manuscript. Janice Doody: Contributed to the data interpretation and revised the manuscript. Shawn Malone: Conceived and contributed to the study design, and revised the manuscript. Eric Vandervoort: Contributed to the study design, collected and interpreted the data, and revised the manuscript.

## CONFLICT OF INTEREST STATEMENT

The authors declare no conflicts of interest.

## ETHICAL STATEMENT

Research Ethic Board protocol: 20180781‐01H.

## Data Availability

Research data are stored in an institutional repository and will be shared upon request to the corresponding author.
